# Implementation of the Care Bundle for the Management of Chronic Obstructive Pulmonary Disease with/without Heart Failure

**DOI:** 10.3390/jcm13061621

**Published:** 2024-03-12

**Authors:** Andrea Bianco, Marco Canepa, Giosuè Angelo Catapano, Maurizio Marvisi, Fabrizio Oliva, Andrea Passantino, Riccardo Sarzani, Paolo Tarsia, Antonio Giovanni Versace

**Affiliations:** 1 Department of Translational Medical Sciences, University of Campania “L. Vanvitelli”, 80131 Naples, Italy; 2 U.O.C. Pneumology Clinic “L. Vanvitelli”, A.O. dei Colli, Ospedale Monaldi, 80131 Naples, Italy; 3 Cardiovascular Disease Unit, IRCCS Ospedale Policlinico San Martino, 16132 Genoa, Italy; 4 Department of Internal Medicine, University of Genova, 16132 Genoa, Italy; 5 G. Monasterio Tuscany Foundation, 56124 Pisa, Italy; 6 Department of Internal Medicine, Cardiology and Pneumology, Istituto Figlie di S. Camillo, 26100 Cremona, Italy; 7 Cardiology 1, A. De Gasperis Cardicocenter, ASST Niguarda Hospital, 20162 Milan, Italy; 8 Division of Cardiology and Cardiac Rehabilitation, Scientific Clinical Institutes Maugeri, IRCCS Institute of Bari, 70124 Bari, Italy; 9 Internal Medicine and Geriatrics, Istituto di Ricovero e Cura a Carattere Scientifico-Istituto Nazionale di Ricovero e Cura per Anziani (IRCCS INRCA), 60126 Ancona, Italy; 10 Department of Clinical and Molecular Sciences, Università Politecnica delle Marche, 60020 Ancona, Italy; 11 Respiratory Unit and Cystic Fibrosis Adult Center, Fondazione IRCCS Ca’ Granda Ospedale Maggiore Policlinico, 20122 Milan, Italy; 12 Internal Medicine Department, Metropolitan Hospital Niguarda, 20162 Milan, Italy; 13 Department of Clinical and Experimental Medicine, Policlinic “Gaetano Martino”, University of Messina, 98100 Messina, Italy

**Keywords:** chronic obstructive pulmonary disease, COPD, exacerbation of COPD, heart failure, differential diagnosis, bundle, NT-proBNP

## Abstract

Chronic obstructive pulmonary disease (COPD) is often part of a more complex cardiopulmonary disease, especially in older patients. The differential diagnosis of the acute exacerbation of COPD and/or heart failure (HF) in emergency settings is challenging due to their frequent coexistence and symptom overlap. Both conditions have a detrimental impact on each other’s prognosis, leading to increased mortality rates. The timely diagnosis and treatment of COPD and coexisting factors like left ventricular overload or HF in inpatient and outpatient care can improve prognosis, quality of life, and long-term outcomes, helping to avoid exacerbations and hospitalization, which increase future exacerbation risk. This work aims to address existing gaps, providing management recommendations for COPD with/without HF, particularly when both conditions coexist. During virtual meetings, a panel of experts (the authors) discussed and reached a consensus on the differential and paired diagnosis of COPD and HF, providing suggestions for risk stratification, accurate diagnosis, and appropriate therapy for inpatients and outpatients. They emphasize that when COPD and HF are concomitant, both conditions should receive adequate treatment and that recommended HF treatments are not contraindicated in COPD and have favorable effects. Accurate diagnosis and therapy is crucial for effective treatment, reducing hospital readmissions and associated costs. The management considerations discussed in this study can potentially be extended to address other cardiopulmonary challenges frequently encountered by COPD patients.

## 1. Introduction

Chronic obstructive pulmonary disease (COPD) is a leading cause of morbidity and mortality worldwide and has the highest rate of hospital admissions among major diseases [1,2]. The high rates of hospitalization due to the acute exacerbation of COPD (AECOPD) represent a substantial economic and social burden, accounting for 50% of the costs of this disease and they are associated with a reduced quality of life and a poor prognosis [2,3,4,5,6,7,8,9,10,11].

AECOPD can be triggered by viral or bacterial respiratory infections [12,13,14,15,16], but environmental factors such as pollution and ambient temperature can increase symptoms [12,17,18,19,20,21,22,23,24,25,26,27,28,29].

Although smoking is a major risk factor for COPD, up to 30% of cases occur in non-smokers [30,31], predominantly women with symptoms of chronic bronchitis [32]. Non-smokers with COPD may be exposed to passive smoke, environmental pollutants, or occupational hazards or have a history of asthma [11]. A recent study showed a link between dysanapsis (a mismatch of the airway tree caliber to lung size) and COPD in older adults, where a lower airway tree caliber relative to lung size increases COPD risk [33]. Non-smokers with COPD exhibit different clinical features [34,35], with predominant airway involvement [34,35] but milder symptoms [31,36,37] and airflow limitation with normal DLCO values [31,36,38,39,40] compared to smokers with COPD. They tend to have a younger age at onset [31] and a poor prognosis with an increased risk of exacerbation [41]. Biomass-exposure-related COPD shows distinct radiological and pathological differences [42], including significant bronchitis and fibrosis [32,43,44]. While pharmacological management is similar for smokers and non-smokers with COPD, individual responses to treatment may vary. Despite limited data, non-smoker COPD is considered a distinct clinical phenotype resembling the chronic bronchitis phenotype.

Several studies have reported that more than 60% of patients with COPD are readmitted to the hospital within 1 year of a previous exacerbation [45,46,47]; recurrent exacerbations and hospitalizations are associated with a poor prognosis because they facilitate a decline in lung function [48] and increase the risk of future admissions [45,46,49,50,51].

The Global Initiative for Chronic Obstructive Lung Disease (GOLD) document classifies COPD based on measured values of FEV1 (forced expiratory volume in 1 s) [11]. The 2024 version of the document emphasizes the role of exacerbations in categorizing COPD patients, using the recently introduced ABE assessment tool, which represents a departure from the earlier ABCD approach [11].

It is important to manage the patient’s comorbidities and identify predictors of hospital readmission [46,52,53] because some of them are not related to COPD. On the contrary, COPD is often part of a more complex cardiopulmonary disease, especially in older patients.

Cardiovascular comorbidities are commonly associated with COPD, making the differential diagnosis challenging [11]. All patients with COPD should have ischemic heart disease taken into consideration and treated, regardless of the presence of concomitant COPD [11]. COPD patients with concomitant ischemic heart disease face a high risk of cardiovascular events within 90 days following an exacerbation [11,54].

Additionally, COPD patients frequently experience cardiac arrhythmias [11,55], and atrial fibrillation is often observed during AECOPD [11,56]. Furthermore, the prevalence of peripheral artery disease is higher in COPD patients compared to the general population and is commonly associated with atherosclerotic heart disease [11,57].

**Figure 1:**
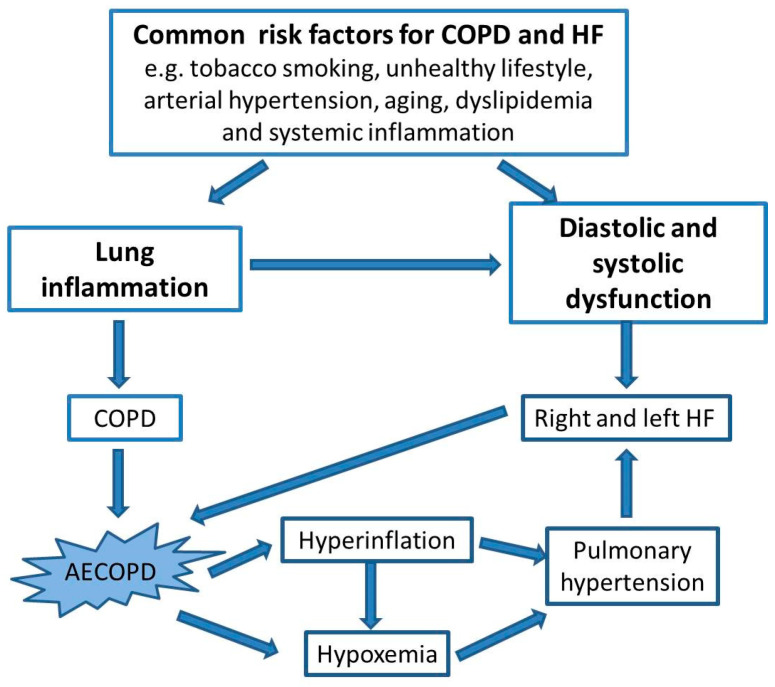
Risk factors shared by COPD and HF. AECOPD: acute exacerbation of COPD; COPD: chronic obstructive pulmonary disease; HF: heart failure.

COPD is a common comorbidity in patients with heart failure (HF); the prevalence of COPD in patients with HF ranges from 20% to 30% [58,59,60,61,62] and a similar prevalence of HF has been reported in large populations of patients with COPD [60,61]. The reasons underlying the high prevalence of COPD among patients with HF and vice versa have long been debated; many studies have shown a contribution of tobacco smoking, because it is a risk factor shared by the two conditions [63,64,65], as well as those of an unhealthy lifestyle, arterial hypertension, aging, dyslipidemia, and the chronic inflammatory burden associated with COPD [60,66,67,68,69,70]. Among the several processes associated with COPD, lung hyperinflation, hypoxemia and systemic inflammation can increase the risk of HF [71]. An overview of the common risk factors for COPD and HF is provided in Figure 1.

COPD worsens the clinical presentation and course of HF and has been associated with a 20–30% increase in the risk of hospitalization of patients with HF and a 1.61× increased risk of mortality [60,72,73,74,75,76,77]. Furthermore, COPD increases the prevalence of comorbidities such as atrial fibrillation, renal dysfunction, diabetes mellitus, higher heart rate and systolic blood pressure, and infection, which can trigger HF decompensation [60,72,75,78]. The 5-year survival rate of patients with HF and concomitant COPD is 31% versus 71% in the absence of COPD [79].

The prevalence of COPD is higher in hospitalized patients with HF [76,80,81] compared with those with chronic HF [60,82,83], probably due to the overdiagnosis of COPD in acute HF settings when symptoms are easily misinterpreted [84]. The differential diagnosis of AECOPD and HF in clinical settings is extremely challenging because they often share a similar clinical presentation with symptoms such as impaired exercise tolerance with exertional breathlessness, fatigue, sleep disturbances such as nocturnal cough and paroxysmal nocturnal dyspnea with no distinctive feature that could help differentiate between COPD and HF [85], muscle weakness, cachexia, and anxiety [72]. AECOPD symptoms such as tachycardia and hypoxemia can precipitate HF decompensation, and pulmonary fluid retention due to HF can worsen airflow restriction [77].

A systematic review found that HF increases hospitalization and mortality in patients with COPD [86], which was corroborated recently in a study of the cause of death among hospitalized patients with AECOPD [74]. The increased risk of death with cardiovascular dysfunction also affects patients with milder or moderate COPD. This was clearly demonstrated in a study by Mannino et al. [87] showing that 27.6% of GOLD 2 patients, 24.8% of GOLD 1 patients, and 39.3% of patients with airway restriction (FEV1/forced vital capacity [FVC] ≥ 0.70 and FVC < 80% predicted) died due to cardiovascular dysfunction, which predominated as a cause of death over lung cancer and respiratory failure.

The GOLD document fails to provide clear indications on the care of patients with COPD/HF, advising to use selective β1-blockers for cardiovascular indications and referring to the HF guidelines [11,88,89,90], which may not be specific enough for these challenging patients [91]. Consequently, patients with known multiple comorbidities such as COPD and HF need a comprehensive health care approach with integrated care provided by pulmonologists and cardiologists [77] rather than the separate treatment of the individual diseases.

A decrease in hospital readmissions needs to be achieved as an outcome in order to improve patients quality of life and to reduce the economic burden related to the disease. Multiple studies dealing with this topic have been published in the literature recently [92,93,94,95,96,97]; in particular, a study by Kalhan et al. [98] proposed a flowchart to simplify and improve the efficacy of the journey for the patient with COPD and HF. A recent review published by Celli et al. [99] provided clear and comprehensive algorithms for the differential diagnosis of a number of clinical conditions that may resemble AECOPD in the acute care setting, including HF.

The aim of the present work is to propose a care bundle to guide clinicians, based on clinical guidelines, on the best practices in the identification, diagnosis, and management of patients with COPD, with or without concomitant HF, with a focus on the optimal practice to distinguish COPD and HF in emergency care settings. As defined by the Institute for Healthcare Improvement, a care bundle is “a small set of evidence based interventions for a defined patient segment/population and care setting that, when implemented together, will result in significantly better outcomes than when implemented individually” [100]. Care bundles have been demonstrated to improve the outcomes of COPD, especially when a respiratory specialist is involved [101,102].

**Figure 2:**
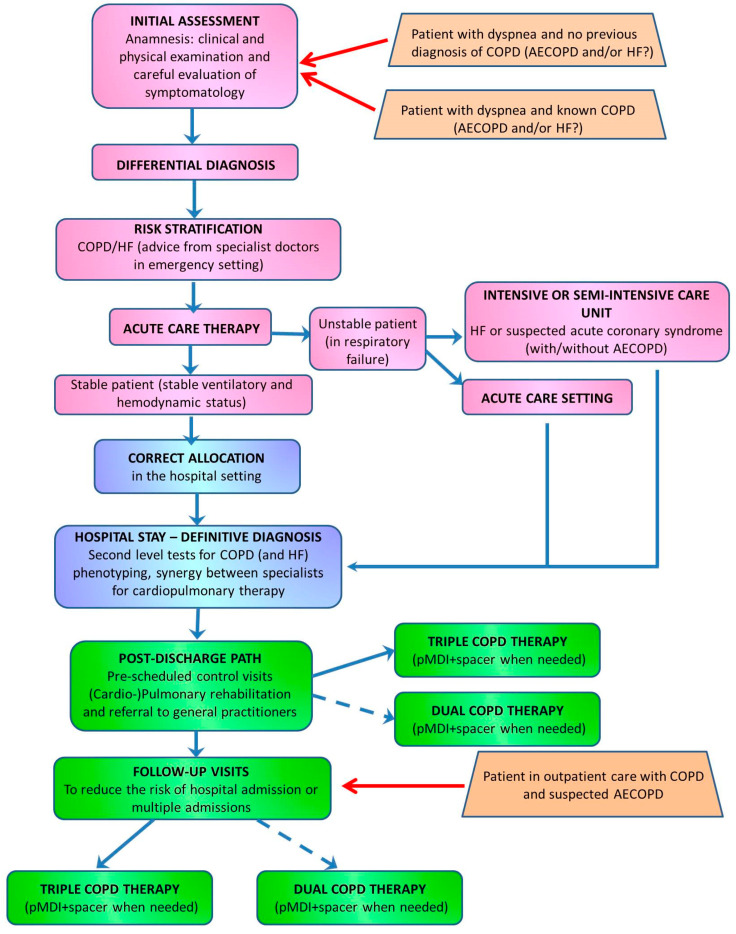
Flowchart summarizing the path for differential diagnosis of COPD and HF. On the left—pink: acute care setting; blue: hospital setting; green: post discharge. On the right—orange: type of patient. AECOPD: acute exacerbation of COPD; COPD: chronic obstructive pulmonary disease; HF: heart failure; pMDI: pressurized metered dose inhaler.

A panel of nine COPD and HF Italian experts, including cardiologists, pneumologists, and internists, participated in a virtual advisory board in October 2022 to provide recommendations on the differential diagnosis of a patient with COPD and risk of HF. The experts provided recommendations for acute care treatment, risk stratification, correct allocation, definitive diagnosis, post-discharge care, and the following up of patients admitted to emergency care settings with dyspnea, with or without a previous diagnosis of COPD and exacerbations. In particular, they highlighted the role of a multidisciplinary approach in the evaluation and management of these patients. They also gave indications on the treatment and following up of patients in outpatient care after an AECOPD event that did not require hospitalization. The recommendations provided by these clinicians to obtain a differential diagnosis of COPD and HF are summarized in a flowchart (Figure 2) and in Supplemental Table S1.

While these experts and their experiences largely reflect the nuances of the Italian health system, the insights could be applicable in part or whole to many other systems managing these patients.

## 2. Differential Diagnosis and Paired Diagnosis

Differential diagnosis has a pivotal role in discriminating between AECOPD and HF in a patient admitted to the hospital due to dyspnea. It guides appropriate acute and chronic therapy and the appropriate location for the patient in the hospital setting. A proper diagnosis reduces the need for unnecessary tests and improves the prognosis, reducing hospital admissions and decreasing costs.

A correct definition of the disease is the fundamental cornerstone of differential diagnosis. The current definition of AECOPD provided by the European Respiratory Society/American Thoracic Society reads as follows: “In a patient with underlying COPD, exacerbations are episodes of increasing respiratory symptoms, particularly dyspnea, cough and sputum production, and increased sputum purulence” [103]. It is similar to the original definition published by René Laennec over 200 years ago [104]. However, both these as well as recent GOLD definitions [11] do not relate the symptoms to measurable variables, and the severity of the event is often related to the medicine used for treatment rather than to the physiologic alterations [105,106]. Accordingly, the underlying triggers of AECOPD are rarely investigated, and the therapeutic options available have not been modified in more than 30 years.

### 2.1. The Importance of a Medical Interview in the Differential Diagnosis

An extensive medical interview that encompasses the known risk factors for COPD plays a fundamental role in the differential diagnosis. A recently published Lancet Commission report on COPD by Stolz et al. [107] highlighted the importance of risk factors, suggesting a classification of COPD into five types based on the risk factors responsible for the etiopathogenesis:

- genetics (i.e., serpin family A member 1 [SERPINA1] gene mutation leading to α-1 antitrypsin deficiency, telomerase reverse transcriptase mutations, or other epigenetic causes still remaining to be defined);
- early life events (i.e., prematurity, low weight at birth, childhood asthma);
- infections (i.e., childhood infections such as with pneumonia and respiratory syncytial virus or adulthood infections such as with tuberculosis and human immunodeficiency virus [HIV]);
- inhalation of tobacco, drugs, and other combustible substances;
- environmental exposures (i.e., indoor pollutants, ambient air pollution, occupational exposures).

The classification suggested in the Lancet Commission has been implemented into the GOLD 2024 report, which proposes a classification into different “etiotypes” reflecting the different pathogenic mechanisms underlying COPD [11].

Accordingly, the clinicians recommend that the medical interview includes information on the risk factors cited above, when available, especially on smoking habits, because tobacco smoke is a leading risk factor for COPD [108]. Additionally, the clinicians advise gathering information on previous spirometry results if available, exacerbation history, and comorbidities (particularly those increasing the overall cardiopulmonary risk, such as hypertension, dyslipidemia, and type 2 diabetes), as well as ongoing pharmacologic and non-pharmacologic therapies.

### 2.2. Differential Diagnosis in the Acute Care Setting: AECOPD and/or HF?

**Figure 3:**
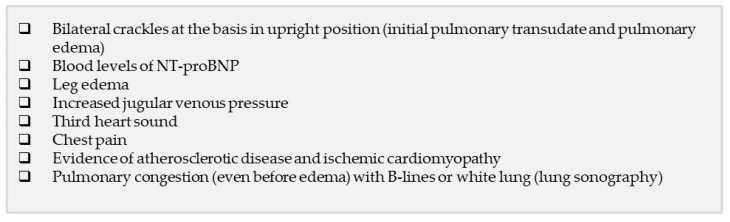
Checklist of the main lab-test, signs and imaging that should lead to suspicion of HF as a cause of or a contributor to COPD.

The differential diagnosis can be extremely challenging in acute care settings, where patients with a previous diagnosis of COPD and patients with symptoms common to COPD and HF who do not have a previous diagnosis are admitted. Accurate clinical history recording and clinical evaluation with proper diagnostic and laboratory tests are the best means of discriminating between COPD and/or cardiovascular dysfunction (Figure 3).

During the evaluation of symptoms and the clinical examination, the authors recommend determining arterial blood pressure, heart and respiratory rate, dyspnea, and sputum production. The respiratory rate is an extremely informative albeit often-neglected measurement, and specific thresholds should be set to increase its clinical significance. A cardiovascular risk score needs to be carefully evaluated if the patient has been hospitalized in an internal medicine setting or in a respiratory ward because COPD must be managed while considering possible underlying cardiovascular dysfunction (i.e., arterial hypertension, ischemic heart disease, and HF). The presence of bilateral crackles at the basis in the upright position (initial pulmonary transudate and pulmonary edema), pulmonary congestion (even before edema) with B-lines, or white lung assessed through lung sonography, leg edema, increased jugular venous pressure, and a third heart sound should guide the diagnosis toward HF as a cause or a contributor to the AECOPD event [109] (Figure 3). The clinical evaluation should collect information about chest pain, signs of atherosclerotic disease, and ischemic cardiomyopathy given the high prevalence of ischemic heart disease in AECOPD events [110,111] (Figure 3). Localized crackles, crepitus at thoracic auscultation, and percussion dullness may indicate pneumonia [99,112], a suspicion that should be accurately ruled out because COPD increases the risk of pneumonia and its severity [113].

The authors recommend several diagnostic tests in an emergency care setting, which are summarized in Table 1.

Among them, blood gas analysis, pulse oximetry, electrocardiography, lung sonography, and chest X-ray should be performed as soon as possible, ideally within the first 24 h after hospital admission. Electrocardiographic monitoring has a strong negative predictive value in ruling out cardiac dysfunction and arrhythmias [114,115] but cannot exclude COPD [116], and pulse oximetry and blood gas analysis, as well as guiding the diagnosis, indicate the severity of the patient’s condition. Chest radiography (upright position) is recommended to exclude diseases such as pulmonary fibrosis, pneumothorax, pneumonia, or lung cancer and to identify signs of HF such as cardiomegaly, pulmonary edema [117], and pleural effusion (Figure 3). The panel strongly emphasizes the need to perform chest radiography in the seated position; most chest radiographs are performed with the patient in a recumbent position due to lack of time and experience, but if the patient has edema caused by congestive HF, this would prevent the proper evaluation of the lungs. Blood gas analysis and chest radiography are also recommended in patients with known COPD who are admitted to the hospital with the need to discriminate between AECOPD or/and underlying HF. Lung sonography should also include the assessment of diaphragmatic excursion; this provides information on respiratory muscle function, especially when blood gas analysis shows an increase in PaCO_2_ (partial pressure of carbon dioxide in arterial blood), and allows the prompt identification of patients with congestive HF (Figure 3). Small portable ultrasonography machines should be available to doctors, and they should be trained in evaluating diaphragmatic excursion. In cases of suspected HF, echocardiography is useful for the evaluation of cardiac structure and function; it should study both the left and the right chambers of the heart, and the left ventricle EF should be evaluated. It can identify pulmonary hypertension by measuring pulmonary artery systolic pressure or pleural effusion. A computed tomography (CT) scan of the chest is recommended for patients who are already diagnosed with COPD and experience multiple exacerbations; in addition, CT identifies opacities that cannot be seen on a chest radiograph [17,118], and it may help discriminate bronchiectasis from AECOPD [11].

The laboratory tests recommended at hospital admission are summarized in Table 1. Venous blood samples should be collected for the following analyses: blood count, erythrocyte sedimentation rate (ESR), C-reactive protein (CRP), platelet count, fibrinogen, D-dimer, troponin, glycemia, creatine kinase muscle–brain isoenzyme (CK-MB), and white blood cell differential, in particular eosinophilic and neutrophilic pattern. Increased CRP levels may indicate bacterial pneumonia, especially if associated with a high white blood cell count [119]. An increased troponin level can be observed in patients with COPD and can increase further during AECOPD [120], but myocardial ischemia should be strongly suspected when associated with an abnormal ECG and symptoms related to cardiac dysfunction [121].

COPD and its comorbidities may account for an additional chronic hemodynamic overloading of the heart. Both B-type natriuretic peptide (BNP) and N-terminal prohormone of brain natriuretic peptide (NT-proBNP) are useful markers to exclude HF in patients with AECOPD [122,123,124,125] (Figure 3), but BNP is less reliable, and the related studies have major limitations such as the underuse of echocardiography to diagnose HF and misdiagnosis of HF due to advanced lung diseases that cause increased pulmonary arterial pressure (cor pulmonale) [126,127]. Natriuretic peptides are commonly increased in patients, with COPD increasing further during AECOPD [128,129,130], so higher thresholds would probably be needed to discriminate between COPD and COPD with concomitant HF, even if abnormalities in other tests may help discriminate. For example, a large pleural effusion—detected through chest radiography, lung sonography, or CT—associated with increased natriuretic peptides guides the diagnosis toward acute HF [99]. Increased natriuretic peptides correlate with an increased risk of death in patients with COPD during AECOPD [131]. European HF guidelines recommend determining blood NT-proBNP levels in patients presenting with signs compatible with HF, including dyspnea, to identify an underlying cardiac involvement that can be further investigated using echocardiography when available [130]. Accordingly, NT-proBNP is a good diagnostic/prognostic marker of heart overload or HF with a high negative predictive value, but it also shows a good positive predictive value in older patients; it is increased in a high percentage of cases of COPD in the elderly, as demonstrated by a retrospective study by Sarzani et al. [132] that included 403 elderly patients (mean age, 88.1 ± 5.1 years) who had been hospitalized for different conditions other than HF. Of the patients with at least one symptom compatible with HF, 61% had NT-proBNP values higher than 1800 pg/mL [132], the most validated and widely used age-adjusted cutoff for a diagnosis of HF [124]. Furthermore, 32.8% of patients had values of NT-proBNP ranging between 300 pg/mL and 1799 pg/mL, which could indicate underlying HF or other types of cardiac dysfunction [132,133]. Of the patients who died during hospitalization, 56.4% had an NT-proBNP value ≥ 1800 pg/mL on admission, and accordingly, high values of NT-proBNP were positively associated with in-hospital death through a logistic regression model [132]. Considering that only 19.7% of patients had a known history of HF, these findings underline the importance of NT-proBNP as a diagnostic criterion for HF in the elderly and also as a prognostic factor for mortality; these results are in line with those obtained in previous studies [134,135,136]. Although European HF guidelines recommend echocardiography to evaluate cardiac structure and function, it is difficult to obtain promptly in emergency settings. The interpretation of its results can be complicated in the elderly due to the high prevalence of atrial fibrillation, which impedes the proper measure of several echocardiographic diastolic components and the uncertain diagnostic values in patients aged >80 years [137,138,139]. NT-proBNP is inexpensive and easy to measure in peripheral blood and should be included in the tests ideally conducted in the first 24 h after hospital admission. Its value as a diagnostic marker of cardiac illness and a prognostic factor for mortality is not influenced by multiple comorbidities in the elderly [140]. Furthermore, its increased levels correlate with a number of echocardiographic abnormalities [139] despite the potential for independently increased levels due to other conditions (e.g., atrial fibrillation, pulmonary hypertension, and right ventricular strain and failure [141,142]).

A consensus document released by the Heart Failure Association of the ESC has explored the practical applications of NT-proBNP across different clinical situations. This research has established validated NT-proBNP cut-off points for non-obese individuals without kidney failure and atrial fibrillation/flutter during the baseline electrocardiogram, stratified based on age and gender, enabling the identification of acute HF in emergency department cases and the diagnosis of de novo HF in outpatient settings [143].

The study also highlights that irrespective of age or gender, patients with NT-proBNP values higher than 2000 pg/mL should undergo echocardiography and clinical evaluation within 2 weeks of diagnosis while HF is very unlikely if NT-pro-BNP values are lower than 125 pg/mL [143].

The assessment of NT-proBNP levels guides the treatment of HF, leading to an increased number of patients treated with β-blockers and renin–angiotensin system blockers, resulting in reduced levels of NT-proBNP at discharge and heart protection [132].

## 3. Acute Care Therapy

If the patient is critically ill, with unstable vital signs, fast action is necessary. Blood gas analysis and electrocardiography are performed and hemodynamic parameters monitored. If acute coronary syndrome is suspected, the patient has to be referred to the cardiac intensive care unit further investigations including a high-resolution CT angiography scan. Blood thinners or thrombolytic drugs are administered in cases of pulmonary embolism.

Treatment is guided by the results of the previous tests; supplemental oxygen is administered if necessary, according to blood gas analysis, and non-invasive ventilation is provided in cases of acute hypercapnic respiratory failure, taking into consideration that oxygen therapy and non-invasive ventilation are most efficacious when started within the first 30 and 60 min, respectively. Non-invasive ventilation is highly recommended for the treatment of AECOPD with acute hypercapnic respiratory failure, as suggested also by the British Thoracic Society guidelines [144], and is frequently used by pulmonologists. During the SARS-CoV-2 pandemic, physicians from different specializations also gained experience in the use of non-invasive mechanical ventilation. Corticosteroids and antibiotics in cases of pneumonia should be administered when indicated. The patient’s health status should be monitored for 6 h and then regularly reevaluated. If HF is present, patients should receive appropriate therapy including a β1-selective blocker, diuretics, angiotensin-converting enzyme (ACE) inhibitors, or, better, angiotensin receptor blockers (ARBs), angiotensin receptor neprilysin inhibitors (ARNIs), and/or sodium–glucose co-transporter 2 inhibitors (SGLT2-is). ACE inhibitors can cause persistent cough and are contraindicated if ARNI use is necessary. If required, dual bronchodilators or triple therapies with a pressurized metered dose inhaler and spacer should be started during the hospital stay for better results as, generally, delayed therapy is related to additional exacerbations and costs [145].

## 4. Risk Stratification and Correct Allocation

Consultation with pulmonologists and cardiologists is fundamental for the correct risk stratification of COPD and/or HF and to avoid misdiagnosis. The patient needs to be referred to the appropriate hospital setting during the hospital stay for proper testing and to reduce the future risk of readmission. After stabilizing the patient with AECOPD, it is important to perform risk stratification for further exacerbations, considering the comorbidities through a synergic interplay between pulmonologists, cardiologists, and internists.

In this context, second-level analyses need to be carried out to provide a definitive diagnosis. Even when the patient is hospitalized in an internal medicine setting, the cardiovascular risk should be assessed. It is necessary to explore both the right and left chambers through echocardiography and to quantify the left ventricular EF to determine whether there is heart dysfunction. Biomarkers such as BNP, NT-proBNP, CRP, and ESR need to be monitored. If hypoxemia is present and its cause is not identified, a pulmonary CT angiography scan and D-dimer analysis may be necessary to exclude pulmonary embolism; when severe hypoxia is present and the patient needs therapy with high-flow oxygen, possibly with pulmonary hypertension as a complication, and COPD and HF are excluded, the scenario is suggestive of pulmonary embolism, which can be difficult to diagnose, especially in older patients with multiple comorbidities.

## 5. Hospital Stay: Definitive Diagnosis

Spirometry evidence of bronchial obstruction is a stringent requirement and a fundamental criterion in pulmonary guidelines to confirm a diagnosis of COPD [11,90]. The authors advise the use of spirometry to discriminate between AECOPD or HF in a patient with previously diagnosed COPD; however, real-world evidence shows that this is not used routinely for COPD patients [146]. During the early 2000s, a significant number of newly diagnosed patients with COPD who met high-risk criteria did not have their diagnoses confirmed through spirometry [146]. However, the situation underwent a rapid change after 2004, with the percentage of patients having a record of spirometry reaching its peak at 72% in 2014 before declining again [146]. By 2019, only 59% of high-risk newly diagnosed patients had a recorded spirometry result within the 12-month period prior to their COPD diagnosis [146].

Spirometry measures lung volumes mobilized with inspiratory or expiratory maneuvers with the aim of determining whether lung obstruction is present. Ventilator defects are common in patients with both HF and COPD, but obstructive spirometry in patients with HF usually improves after the use of diuretics [147,148]. However, spirometry is rarely considered in patients with HF and known or suspected COPD [60]. Canepa and colleagues calculated that only 19.6% of patients hospitalized with HF and 30.6% in a population of patients with chronic HF underwent spirometry [75,91]. In a cohort study, it was found that 65% of COPD patients with concomitant HF receiving appropriate HF therapy and 39% of COPD patients receiving inadequate HF therapy underwent spirometry testing [149]. Additionally only 35% of patients with both HF and newly diagnosed COPD, who were prescribed inadequate therapy for COPD, had recorded spirometry results [149]. Conversely, among HF patients who were prescribed appropriate therapy for newly diagnosed COPD, the percentage of recorded spirometry results increased to 57% [149]. A possible explanation for the underuse of spirometry in patients with HF who have symptoms of COPD is that spirometry is not easily accessible to cardiologists and the fact that the cardiology community shows some skepticism towards the discriminatory capacity of spirometry in patients with HF [72]; accordingly, the prevalence of COPD is notably lower in patients managed by cardiologists as opposed to general physicians [150,151,152,153]. The interpretation of spirometry results during acute decompensation in patients with HF can be challenging because “wet lung HF” may present with airway restriction or obstruction [62], so the current guidelines of both the European Society of Cardiology [154] and the GOLD group [11,90] recommend that spirometry is performed when HF conditions are stable [62,75,148,155]. However, timely referral and following up to the pulmonologist or respiratory clinic, or alternatively the use of spirometry in cardiac clinics, needs to be implemented because its scarce use leads to about 30% of patients with chronic HF being mislabeled with COPD without confirmation of airflow obstruction, and a similar proportion of patients have unrecognized airflow obstruction [156]. A restrictive spirometric pattern warrants further investigation to determine whether it indicates HF decompensation, airway wall edema due to congestion, other pulmonary conditions, or neuromuscular disease [157].

The GOLD and American Thoracic Society/European Respiratory Society guidelines defined a fixed cutoff for the ratio of post-bronchodilator FEV1 to an FVC [158] of <0.70 [11,159] for the diagnosis of persistent airflow reduction and the use of the percentage of predicted FEV1 to classify the degree of obstruction [90]. The fixed cutoff value of FEV1/FVC < 0.70 may lead to the overdiagnosis of COPD in elderly patients because this ratio decreases with aging [160]. An alternative approach was proposed whereby airflow limitation is diagnosed when the FEV1/FVC is lower than or equal to the lower limit of normality (LLN), which is the fifth percentile of a healthy, nonsmoking population [160]. This latter option may be more accurate, but sometimes, patients may have a normal spirometry considering the LLN and an abnormal spirometry using the fixed cutoff; an increased risk of hospitalization in this subgroup of patients has been reported [160] and since the fixed ratio is simpler and more consistent to use than LLN, the international guidelines for COPD promote the use of the fixed ratio over the LLN [11,90].

Dyspnea is a common presentation of decompensated HF, COPD, or asthma. Differentiating AECOPD from asthma is another clinical challenge: asthma and COPD can be comorbid [161], symptoms often overlap, and even though reversibility with bronchodilators [162] and resolution with inhaled corticosteroids [90] are suggestive of asthma, there are exceptions. In addition to spirometry, other tests such as of the diffusing capacity of the lungs for carbon monoxide (DLCO) and bronchial provocation testing may help diagnose asthma. However, differences between COPD and asthma are beyond the scope of this paper; for more details, we refer the reader to extensive reviews of the literature on this topic [163,164,165].

Diagnostic tests such as spirometry, DLCO, and cardiac biomarkers such as NT-proBNP, echocardiography, and chest imaging are fundamental to diagnose HF in patients with COPD and to discriminate between AECOPD and HF in patients admitted to the hospital with dyspnea. The same synergistic approach should be used to differentiate COPD from other conditions that have overlapping symptoms, such as asthma, bronchiectasis, pneumonia, and pleural effusion, as highlighted by the recent review published by Celli and colleagues [99]. Past history and clinical evaluation should be combined with the results of imaging and laboratory tests and with spirometry. A synergistic approach that involves cardiologists, pulmonologists, and internists is fundamental to interpret the results. The British Thoracic Society recommends a specialist examination within the first 24 h of hospital admission. Although this can be difficult to obtain in most care settings, at least in Italy, a specialist visit before hospital discharge is highly advised for all patients admitted for AECOPD to provide a definitive diagnosis.

## 6. Hospital Discharge and Follow-Up

The correct planning of follow-ups and post-discharge care can have an enormous impact on reducing hospitalizations; several previous studies have demonstrated the beneficial effect of timely follow-up visits and disease-specific management programs [166,167,168,169]. These may consist of medical education sessions on COPD (to increase knowledge of the disease and treatment adherence); demonstrations of inhaler techniques; discharge planning of an appropriate maintenance treatment; counseling to quit tobacco smoking; and recommendations to refer to pulmonary rehabilitation programs, to adhere to influenza and pneumococcal vaccination programs, and to exercise regularly, as well as coaching by a case manager [6,170,171]. These recommendations have all been shown to be useful in hastening recovery, reducing hospitalizations and readmissions [166,167,168,169,170,172,173,174,175,176,177,178], and reducing the economic burden for each patient [179]. Recommendations for patients and caregivers are summarized in the Supplemental Table S2. The transition from hospital to home or to long-term care facilities has a fundamental role for a successful management of COPD after discharge, and it should be handled considering care coordination and a structured discharge planning.

In a recent study, COPD patients participated in a video telehealth-based pulmonary rehabilitation program comprising 36 exercise and educational sessions over 12 weeks, customized to their individual baseline characteristics [180]. The findings revealed a decrease in the 30-day all-cause readmission rate, a reduction in the 30-day readmission rate specifically due to AECOPD, and an extended time to the first readmission in the telehealth groups compared to the control group [180]. Support at home with telemonitoring may reduce mortality and readmissions after 12 months, as suggested by a study conducted by Marcos et al. [181]. A recent systematic review reported a decrease in readmission rates and/or an improved emotional state in COPD patients provided with personalized case management action plans with constant phone calls, visits, and technical training and education after discharge [182]. Additional research is necessary to underscore the advantages and the great potential linked to telehealth-based interventions [183], as the diversity in clinical trials currently hinders a comprehensive understanding of their potential to enhance COPD healthcare [184,185,186].

The main care gaps in the management of patients with COPD that are responsible for the increase in readmission rates are significant variability in inpatient care and suboptimal care transitions such as a lack of access to timely follow-ups and early disease management programs [9,10,90,172,173,187,188,189,190,191,192,193,194,195,196,197]. Care gaps post discharge are particularly significant for patients with COPD; in some studies, more than 50% of incidences of AECOPD were not reported to health care providers, with a significant impact on health status [51,174,198]. A coordinator should be in charge of managing a structured program for the discharge of COPD patients, alleviating their burden and responsibilities [182]. The CONQUEST multi-national program developed quality standards for the primary and secondary care of COPD patients [199]. These standards aim to promote early intervention in identifying, assessing, and effectively managing both diagnosed and undiagnosed COPD patients who are at a modifiable and high risk of exacerbations [199]. Furthermore, the program emphasizes the importance of follow-up care for such patients [199].

A study conducted two years later compared the management of high-risk COPD patients in the UK with national and international management recommendations and quality standards including those of the CONQUEST program [146]. The study revealed that there is a significant oversight in identifying COPD patients at high risk of exacerbations early on [146]. Both newly diagnosed and already-diagnosed patients at high risk are not promptly assessed or treated [146]. This highlights a considerable opportunity for enhancing the assessment and optimization of treatment for these patients.

COPD tends to be undervalued by primary care physicians compared with other diseases such as cardiovascular disorders, probably because of the absence of specific biomarkers. It is necessary to inform primary care physicians about the need for the punctual following up of patients with COPD; they must monitor the risk of exacerbation after hospital discharge, adherence and response to treatment, and the symptom burden, especially during the first month after discharge when the risk of cardiovascular or ischemic attacks is high, and they should ensure appropriate home assistance if needed. The authors recommend a follow-up visit scheduled up to 12 weeks after discharge if the patient was admitted in an internal medicine setting or 1 month after discharge if the reason for hospital admission was acute HF. A post-discharge follow-up visit within the first 30 days has been shown to significantly reduce mortality in patients with COPD [200]. The authors underline that scheduling visits 6 months or more after hospital discharge for the last event of AECOPD is not useful because the risk of additional exacerbations is higher in the first months.

The visit should take place in pulmonology, cardiology, or internal medicine units according to the patient’s diagnosis. During the visit, it may be useful to perform blood gas analysis, spirometry, and the 6 min walking test and to consult the pulmonologist in case of latent respiratory failure. Additional parameters such as vital signs, pulse oximetry, renal function, and blood inflammatory markers could also be assessed.

## 7. Post-Discharge COPD and COPD/HF Therapy

The recently updated GOLD document for the treatment of COPD [11] recommends the following:

- Patients with COPD who present with occasional symptoms or are already under treatment with long-acting bronchodilators but need immediate relief of symptoms are treated with short-acting bronchodilators: either a short-acting muscarinic antagonist or a short-acting β2-agonist or their combination [11].
- Patients with COPD with dyspnea and two or more moderate AECOPDs or with one or more severe AECOPDs requiring hospitalization are treated with a long-acting β2-agonist (LABA) or a long-acting muscarinic antagonist (LAMA) or their combination; triple therapy with LABA + LAMA + inhaled corticosteroids (ICSs) is recommended if the eosinophil count is above the threshold of ≥300 eosinophils/μL [11]. However, eosinophil levels should be combined with clinical assessment as certain patients with eosinophil counts below 300 eosinophils/μL could also benefit from triple therapy [11].
- Patients with COPD with no or one moderate AECOPD and without the need for hospitalization should be treated with a single bronchodilator (mMRC score 0–1, CAT score < 10) or with LABA + LAMA (mMRC score ≥ 2, CAT score > 10). When patients experience recurrent exacerbations despite treatment with a dual bronchodilator (LABA + LAMA), the GOLD guidelines recommends switching to triple inhaled therapy with LABA + LAMA + ICSs [11].

If there is an indication for the use of ICSs, the new 2024 GOLD document recommends triple inhaled therapy with LABA + LAMA + ICSs over LABA + ICS therapy [11] according to the results from recent ETHOS and IMPACT trials, which suggest a reduction in mortality among patients with COPD with the use of triple inhaled therapy compared with dual therapy (LABA + ICS) [201,202].

Although definite indications exist for COPD treatment and management, no recent guidelines exist for the treatment of patients with concomitant COPD and HF, and therefore, many patients do not receive appropriate treatment. The cohort study conducted by Kostikas et al. [149] revealed that among a total of 12,587 patients who had both COPD and newly diagnosed HF, adequate HF therapy was prescribed for 18% of the patients. Inadequate therapy for HF was prescribed for 42% of the patients, and a significant portion (40%) remained untreated for HF [149]. Consistent with these results, Canepa et al. highlighted, in their review on the diagnostic and therapeutic gaps in patients with COPD and HF, that half of these patients are not treated with any β-blocker when they are admitted to the hospital and only one third of them are discharged with a β-blocker prescription [91]. The underuse of β-blockers has been associated with increased mortality [203], and there is no rationale for withholding them from patients with COPD/HF [204] because COPD is not a contraindication to β-blockers [154,205] and asthma is only a relative contraindication; in fact, β-blockers seem to improve survival in patients with COPD who do not have HF [203]. Based upon a Cochrane review, the use of β1-selective blockers (i.e., nebivolol and bisoprolol) is preferred and recommended in patients with chronic HF and COPD because they are associated with a lower risk for bronchoconstriction [206]. The reason underlying the low prescription rates of β-blockers for patients with COPD/HF seems to be due more to clinician inertia rather than to a lack of patient compliance [91,207]. SGLT2-is may represent a valid option for the treatment of patients with HF in all the EF ranges (HFrEF, HFmrEF, HFpEF) and concomitant COPD as an additional treatment or, occasionally, as an alternative therapy for patients who are intolerant to β-blockers, as demonstrated in a study by Dewan and colleagues, which showed that dapagliflozin, an SGLT2-i, had a favorable effect in a population of patients with HFrEF and COPD [208,209]. In all cases, optimal blood pressure control must be pursued using combinations of renin–angiotensin–aldosterone inhibitors (RAASis) and appropriate diuretics, especially in people with a tendency for salt and water retention. RAASis are also recommended in patients with HFrEF. ARNIs can also be indicated and ARBs should be preferred before their use, also to avoid confusion from ACE-inhibitor-induced cough. The glucagon-like 1 peptide receptor agonist (GLP-1 RA) might have an increasing role in obese COPD patients, especially if there is concomitant HF. The serial testing of NT-proBNP levels should be included in the post-discharge indications for follow-ups due to its potential usefulness in titrating the therapy for HF [210,211]; the optimal monitoring interval needs to be tailored to the underlying risk in the patient [212]. The attention from the cardiology community needs to greatly improve regarding the consequences of COPD as a dangerous comorbidity of HF and its relationship to other cardiopulmonary conditions. All these recommendations are especially important in older patients.

## 8. Outpatient Care

In cases of AECOPD not requiring hospitalization, appropriate outpatient care is also fundamental for the optimal management of the disease to reduce the risk of AECOPD and future hospital admissions. Several studies have demonstrated that each exacerbation increases the risk of future exacerbations in a graduated fashion [213,214,215,216] and that the grade of severity of each exacerbation increases the risk of death [215]. The effective prevention of further AECOPD occurrences relies on the timely management of exacerbations through appropriate therapy. The EROS real-world retrospective study demonstrated that initiating triple therapy with budesonide/glycopyrronium/formoterol fumarate within 30 days of a moderate or severe exacerbation in COPD patients is associated with a reduced risk of future exacerbations [217]. The study revealed a 24% decrease in exacerbation risk compared to delaying treatment by one to six months and a 34% decrease compared to delaying treatment for six months to one year [217].

Exacerbations increase the cardiovascular risk in COPD patients, as demonstrated by a study on 25,857 patients with COPD [218]. The study showed that the exacerbation increases the risk of myocardial infarction 2.7-fold and the risk of stroke 1.26-fold 1–5 days and 1–49 days after exacerbation, respectively in patients without a previous history of cardiovascular abnormalities [218]. According to a retrospective cohort study involving 355,978 patients with COPD, the occurrence of a single acute moderate or severe COPD exacerbation was found to increase the risk of an acute severe cardiovascular event by 32% within the first 30 days compared to individuals without a prior COPD exacerbation [219]. The study also revealed that the risk of cardiovascular events was most pronounced during the initial 90 days following the COPD exacerbation, and it remained elevated for a duration of one year [219]. Furthermore, each subsequent COPD exacerbation was associated with an even higher risk of acute cardiovascular events [219].

A phase 3 randomized, double-blind, 52-week trial comparing fluticasone furoate, umeclidinium, and vilanterol in different combinations in patients with COPD demonstrated an increased risk of cardiovascular adverse events, hospitalization, or death during moderate or severe AECOPD, which decreased over time [220]. Other risk factors for HF in patients affected by COPD are summarized in Figure 1.

Hospital admissions markedly increase the risk for death, as demonstrated by a study on a large population-based inception cohort of 73,106 patients with COPD with long-term follow-ups; 50% of patients with COPD died within 3.6 years after the first hospitalization and 75% within 7.7 years [213].

For these reasons, it is paramount that outpatient care provides an evaluation of COPD and cardiovascular risk. Each patient who accesses an outpatient facility with symptoms potentially caused by COPD and/or HF should receive an accurate risk stratification and be treated appropriately in relation to the identified condition. Clinicians should remember that the ultimate aim of outpatient care is to avoid exacerbations and hospital admissions. A true commitment of outpatient care to adequately treat patients with COPD, both with and without concomitant HF, with a synergistic approach by pulmonologists and cardiologists, with the monitoring of pulmonary and cardiac functions (including, if possible, serial testing of NT-proBNP levels to identify patients at high risk who need a modification of the therapy) at each visit and an appropriate use of β1-blockers when necessary would greatly reduce the risk for AECOPD and, as a consequence, the need for hospital admissions and the risk of death.

## 9. Conclusions

In this article, the authors have underlined several aspects that can help differentiate between COPD and HF, with a special emphasis on the need to differentiate between dyspnea due to COPD and due to HF in its different forms. At the same time the authors have underlined the importance of both diagnoses because COPD and HF often coexist, especially in older patients. To this end, the authors have provided a list of recommended laboratory and diagnostic tests, highlighting the value of NT-proBNP levels, the use of spirometry, and the importance of performing chest radiography in a seated position to rule out confounding factors due to pulmonary congestion and/or edema. Lung sonography also has an increasing role in internal medicine.

The authors have proposed an algorithm to improve the management of hospitalized patients, but greater efforts should be directed towards preventing hospitalizations due to AECOPD because they markedly increase the risk of future hospitalized exacerbations, cardiovascular complications, and death. Outpatient care plays a fundamental role as proper outpatient management could prevent hospital admissions of patients with COPD who have never been hospitalized before, greatly improving their prognosis. In this setting, the appropriate evaluation of global cardiovascular risk and appropriate treatment to reduce it are mandatory in patients with COPD who are often elderly and have several cardiovascular risk factors. The authors have also highlighted the need to treat COPD and HF appropriately even when they coexist, underlining the fact that selective β1-blockers, currently underused in patients with COPD and concomitant HF, are beneficial in these patients. While the connection between COPD and HF is the focus, COPD patients may have several other cardiopulmonary comorbidities and risks to manage; several recommendations (e.g., spirometry, cardiac risk assessment, etc.) could be applicable to these other conditions as well. The diagnostic and therapeutic path illustrated in this article may help avoid misdiagnosis and achieve an overall reduction in hospital readmissions, with better prognosis for patients and reduced costs related to COPD, especially when it coexists with HF.

## Figures and Tables

**Table 1:** Clinical evaluation recommended in the acute care setting for a patient with AECOPD and/or HF.

**Laboratory Tests**	**Imaging**
Blood cell count Plasma electrolytes	Chest radiography
ESR	Lung sonography with assessment of diaphragmatic excursion and pleural effusion
CRP	Chest computed tomography scan
D-dimer	**Diagnostic tests**
	Electrocardiography
hs-cardiac troponin	Pulse oximetry
Glycemia	Blood gas analysis
Eosinophilic and neutrophilic pattern	
NT-proBNP (recommended both as a diagnostic/prognostic test for heart overload or HF or to exclude them)	

ESR: erythrocyte sedimentation rate; CRP: C-reactive protein; hs-troponin: high-sensitivity troponin; NT-proBNP: N-terminal prohormone of brain natriuretic peptide; HF: heart failure.

## Data Availability

No new data were created or analyzed in this study. Data sharing is not applicable to this article.

## Supplementary Materials

The following supporting information can be downloaded at: https://www.mdpi.com/article/10.3390/jcm13061621/s1, Table S1: Management recommendations for clinicians at different stages of COPD patient journey; Table S2: COPD management recommendations for patient/caregiver.
