# Supplementary material for: Implementation of the Care Bundle for the Management of Chronic Obstructive Pulmonary Disease with/without Heart Failure
Source: J Clin Med. 2024 Mar 12;13(6):1621. doi: 10.3390/jcm13061621 (PMC10971568; doi:10.3390/jcm13061621)
Supplement: Supplementary file 1 [file jcm-13-01621-s001.zip › jcm-2878526-supplementary.pdf]

Supplemental Table S1. Management recommendations for clinicians at different stages of COPD patient journey.

**Initial assessment**	
Anamnesis	Focus on COPD risk factors
Evaluation of symptoms and clinical examination	Evaluation of arterial blood pressure, heart and respiratory rate, dyspnea and sputum production<br>Careful evaluation of cardiovascular risk score
Diagnostic tests in emergency setting	Blood cell count; plasma electrolytes; ESR; CRP; D-dimer; hs-cardiac troponin; glycemia,; eosinophilic and neutrophilic pattern; NT-proBNP; chest radiography; lung sonography; chest computed tomography scan; electrocardiography; pulse oximetry; blood gas analysis.
**Differential diagnosis**	
COPD/HF	Bilateral crackles at the basis in upright position; NT-proBNP; leg edema; increased jugular venous pressure; third heart sound; chest pain; evidence of atherosclerotic disease and ischemic cardiomyopathy; pulmonary congestion (even before edema) with B-lines or white lung (lung sonography).
COPD/pneumonia	Localized crackles<br>Crepitus at thoracic auscultation<br>Percussion dullness
COPD/asthma	Check reversibility with bronchodilators and resolution with inhaled corticosteroids<br>In case of doubt perform DLCO and bronchial provocation testing
**Acute care therapy**	
Correctly allocate patients (unstable patients: intensive or semi-intensive care unit or acute care; stable: hospital ward	
Administer blood thinners or thrombolytic drugs in case of pulmonary embolism	
Supplement oxygen according to blood gas analysis results within 30 minutes	
Provide non-invasive ventilation in case of acute hypercapnic respiratory failure within 60 minutes	
Administer corticosteroids and antibiotics in cases of pneumonia	
Administer anti-hypertensive treatment, including β1-selective blockers, if HF is present	
Monitor patient's health status for 6 hours and then regularly	
Start dual bronchodilators or triple therapies with a pressurized metered dose inhaler and spacer	
**Hospital allocation**	
Consult with pulmonologists and cardiologists to refer the patient to the appropriate hospital setting	
Perform echocardiography, and evaluate left ventricular EF and the right heart (e.g. tricuspid annular plane systolic excursion (TAPSE))	
Monitor NT-proBNP (or BNP), CRP, and ESR levels	
In case of hypoxemia, consider a pulmonary CT angiography scan and D-dimer analysis to exclude pulmonary embolism	
**Definitive diagnosis**	
Perform spirometry for definitive diagnosis (when HF condition is stable)	
In case of restrictive spirometric pattern further investigate	
**Hospital discharge and follow-up/Outpatient care**	
Program timely follow-up visits	
Provide personalized disease-specific management programs	
Schedule a follow-up visit up to 12 weeks or 1 month after discharge if the patient was admitted in an internal medicine setting or due to acute HF, respectively	
During the follow-up visits perform blood gas analysis, spirometry and the 6-minute walking test, assess vital signs, pulse oximetry, renal function, and blood inflammatory markers and consult the pulmonologist in case of latent respiratory failure	
Assess NT-proBNP levels at follow-up visits to titrate HF therapy	
**Post-discharge COPD and COPD/HF therapy**	
Do not withhold β_1-selective blockers in case of concomitant COPD/HF	
Control hypertension with appropriate therapy (RAASi and appropriate diuretics and/or calcium channel blockers)	
Assess and optimize inhaler device technique	

COPD: chronic obstructive pulmonary disease; HF: heart failure; ESR, erythrocyte sedimentation rate; CRP, C-reactive protein; DLCO: diffusing capacity of the lungs for carbon monoxide ; ACE: angiotensin-converting enzyme; EF: ejection fraction; BNP: B-type natriuretic peptide; N-terminal prohormone of brain natriuretic peptide; CRP: C-reactive protein; ESR: erythrocyte sedimentation rate; RAASi: renin-angiotensin-aldosterone system inhibitors.

Supplemental Table S2. COPD management recommendations for patient/caregiver

Management of:	
Therapy	Recommend continuation of COPD therapy also in the absence of respiratory symptoms
	Follow the instructions provided for the correct use of the respiratory devices
	Follow the instructions provided for a correct use of the pressurized metered dose inhaler and spacer
Symptoms	Be aware that dyspnea may result from not only COPD but also other comorbidities like HF
	Adopt a daily routine of moderate physical exercise
Risk factors	Reduce or abolish risk factors (tobacco smoke, air pollution)
	Adhere to recommended vaccines

COPD: chronic obstructive pulmonary disease.
